# Single-cell transcriptomics refuels the exploration of spiralian biology

**DOI:** 10.1093/bfgp/elad038

**Published:** 2023-08-22

**Authors:** Laura Piovani, Ferdinand Marlétaz

**Affiliations:** Centre for Life’s Origins and Evolution (CLOE), Department of Genetics, Evolution & Environment, University College London, Gower Street, London, UK; Centre for Life’s Origins and Evolution (CLOE), Department of Genetics, Evolution & Environment, University College London, Gower Street, London, UK

**Keywords:** scRNA-seq, spiralia, Lophotrochozoa, larva, regeneration, brain

## Abstract

Spiralians represent the least studied superclade of bilaterian animals, despite exhibiting the widest diversity of organisms. Although spiralians include iconic organisms, such as octopus, earthworms and clams, a lot remains to be discovered regarding their phylogeny and biology. Here, we review recent attempts to apply single-cell transcriptomics, a new pioneering technology enabling the classification of cell types and the characterisation of their gene expression profiles, to several spiralian taxa. We discuss the methodological challenges and requirements for applying this approach to marine organisms and explore the insights that can be brought by such studies, both from a biomedical and evolutionary perspective. For instance, we show that single-cell sequencing might help solve the riddle of the homology of larval forms across spiralians, but also to better characterise and compare the processes of regeneration across taxa. We highlight the capacity of single-cell to investigate the origin of evolutionary novelties, as the mollusc shell or the cephalopod visual system, but also to interrogate the conservation of the molecular fingerprint of cell types at long evolutionary distances. We hope that single-cell sequencing will open a new window in understanding the biology of spiralians, and help renew the interest for these overlooked but captivating organisms.

## INTRODUCTION

**Figure 1:**
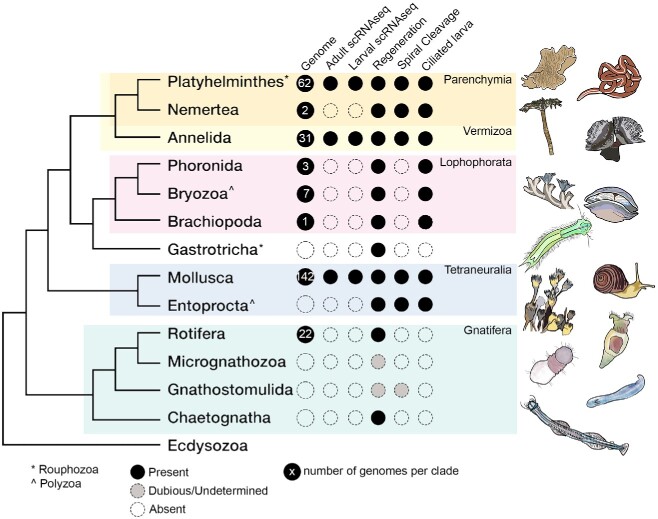
A tree of Spiralia [11] showing shared characters and resources currently available. The asterisk (^*^) indicates the clades that belong to the alternative Rouphozoa clade and those marked by (^) to the Polyzoa clade. Genome numbers per clade are retrieved from NCBI by selecting scaffold minimum assembly, one per species.

The establishment of Spiralia as one of the three main bilaterian superclades together with Ecdysozoa and Deuterostomia was a major achievement of molecular phylogeny [1, 2]. Spiralia is one of the most diverse groups of animals, ranging from microscopic organisms living on the mouthpiece of lobsters, to the exquisite complexity of cephalopods and their advanced behaviour and giant worms extracting their subsistence from hydrogen sulphide through symbiosis. But they also remain one of the most enigmatic, in many ways. Broad aspects of the biology of many spiralian groups remain poorly understood, particularly at the molecular level. One of the reasons is that most of what we know about biological functions is derived from the laboratory study of a handful of model species that belong either to deuterostomes (mouse, zebrafish and, of course, human) or ecdysozoans (fruit fly, nematode). For instance, in the curated swissprot database, only 0.47% of the proteins come from spiralians [3], and spiralian protein structure is known for only 0.34% records in the Protein Data Bank database [4]. The first spiralian genomes were only described in 2013 for annelids and molluscs [5] and despite the tremendous progress in genome sequencing, no genome has been yet released for five spiralian phyla (Figure 1) [6–9].

Moreover, the internal relationships of spiralians remain disputed, despite the availability of transcriptome and even genomic data in more and more lineages [10–12], particularly, because several clades (gastrotrichs, gnathostomulids, platyhelminthes) are prone to long-branch attraction due to their fast rates of evolution [13]. Recently, the clade Gnathifera, gathering chaetognaths, rotifers and other jaw-bearing groups, has been established [11, 12]. The relationships among lophotrochozoans taxa however remain contentious, particularly that of annelids, molluscs, platyhelminthes and lophophorates (Figure 1). In this review, we refer to the sister-group of Gnathifera as Lophotrochozoa for convenience and clarity, but we acknowledge that some authors might hold other views [14]. For instance, platyhelminthes have moved from representing a relatively early diverging lineage among spiralians together with gastrotrichs (the ‘Rouphozoa’ hypothesis [15]) to possibly be close relatives to nemerteans and annelids (the ‘Vermizoa’ clade [11]) (Figure 1). Similarly, the monophyly of lophophorates has been repeatedly questioned and reaffirmed, with bryozoans sometimes excluded from it [10–12]. Interestingly, aforementioned clades such as Vermizoa or Rouphozoa are not supported by many morphological and embryological characters, if any. The two main unifying characters of spiralians are the presence of spiral cleavage that goes through a stereotyped pattern of early cell division with a 45° tilt angle [16, 17] and the occurrence of a ciliated larval form, the trochophore larva present for instance in mollusc and annelids, whose homology is still debated [18, 19]. At the same time, spiralian phyla conceal one of the most astonishing diversity of organisms and lifestyle, and many of their novelties and innovations remain poorly characterised at the molecular level because of the lack of spiralian genetic models. Among such novelties, one could mention the molluscan shell, the brain and sensory organs of cephalopods and the advanced regenerative abilities found in flatworms. Some of these characters have not yet been explored at the molecular level: does gene expression conservation correlate with morphological similarities?

Single-cell technologies enable the characterisation of gene expression (and more recently of chromatin occupancy) at the individual cell level [20], which makes them very powerful tools to classify cell types or to reveal concealed cell type heterogeneity [21]. This approach has made it possible to characterise the cell type complement in cnidarians, ctenophores and sponges [22–24], which highlighted the conserved role of transcription factors (TFs) in establishing metazoan cell types. Although deploying single-cell sequencing in marine invertebrates can represent a challenge, it provides an unparalleled ability to characterise the molecular diversity in a new lineage, giving insight into metabolism, pathways or structural proteins, especially when cell types are traced into the organism using advanced *in situ* gene expression and imagining, such as hybridisation chain reaction [25].

In this review, we will survey the recent progress in exploring cell type evolution in spiralians (Table 1), and their impact on our understanding of their biology and phylogeny.

**Table 1:** Details of single-cell transcriptomic atlases generated for spiralian clades to date.

**Group**	**Species**	**Tissue**	**Reference**	**Technology**	**Cell number**
Platyhelminthes	*S. mediterranea*	A	[26–29]	Drop-seq, ACME+Split-seq	50,562—21,612—19,025—2,000
	*D. japonica*	C, A	[30, 31]	Ad hoc, ACME+Split-seq	~550—13,406
	*S. mansoni*	L	[32–34]	10×G, Smart-seq2	3,226
	*P. crozieri*	L	[18]	10×G	17,605
Annelida	*C. teleta*	L	[35]	10×G	2,857
	*P. dumerilii*	L	[36]	Fluidigm	373
	*E.andrei*	O, A	[37, 38]	10×G, Microwell-seq	2,080—95,020
	*P. leidyi*	A	[39]	ACME+Split-seq	75,218
Mollusca	*C. hongkongensis*	C, A	[40]	10×G	4,639
	*C. gigas*	L	[18]	10×G	8,597
	*D. rostriformis*	L	[41]	10×G	632
	*L. vulgaris*	O	[42]	10×G	19,974
	*E. berryi*	O	[43]	10×G	98,537
	*O. bimaculoides*	O	[44]	10×G	28,855
	*O. vulgaris*	O	[45]	10×G (cells and nuclei)	~17,000
A= adult	L= larva	C= subset of cells	O = subset of organs	10×G = 10× Genomics	

### Single-cell and spiralian larvae

Ciliated larvae, such as the iconic trochophore larva, are present in 8 out of the 13 spiralian clades and in almost all phyla of the clade Lophotrochozoa (Figure 1), but their potential homology has been hotly debated. Spiralian larvae all display one or more bands of ciliated cells that they use for swimming, an apical organ connected to a ciliary apical tuft that has a sensory role, paired protonephridia for excretion, a larval gut and sometimes larval eyes. Historically, it was proposed that the larva of annelids and molluscs—called the trochophore—was the ancestral larva of Spiralia and of protostomes [26–28]. However, more recent molecular phylogenies do not necessary recover a close association of molluscs and annelids [11, 12], which led to question the homology of trochophore larvae and of other ciliated larvae of spiralians [18, 19].

In recent years, transcriptomic tools have been used to try and tackle this long standing debate, for instance, by monitoring the age of genes expressed during embryonic and larval development in molluscs [29–31]. These analyses revealed a peak of expression of novel genes at the larval stage compared to other embryonic stages, thus challenging the idea that trochophore larvae could represent the ancestral larval type.

Conversely, a comparison of gene expression and gene regulation through larval and juvenile development of several annelids showed that indirect developers shift the activation of posterior genes to the onset of metamorphosis [9]. As similar mechanisms of delayed trunk development are observed across Bilateria, this led to the suggestion that the regulatory mechanisms operating to modulate larval stages are universal and could be ancestral [32, 33].

More recently, single-cell transcriptomics was proposed as a new source of evidence to assess whether spiralian larvae are homologous by cataloguing their cell type complement and comparing their transcriptomic signatures. Currently, several explorative scRNA-seq datasets (i.e. with low cells/genes number) of larvae have been collected in molluscs and annelids (See Table 1). We attempted a more ambitious comparison between the trochophore larva of the Pacific oyster and the Mülller larva of polyclad flatworms by sampling larger datasets and applying explicit comparative methods [18, 34]. Single-cell sequencing of the oyster larval stage highlighted that the expression of novel genes is restricted to shell gland cell types [18]. This finding refutes the hypothesis of a recent origin of trochophore larvae and highlights the strength of single-cell transcriptomics in clarifying patterns of evolution of novelties.

In this study, we also detected conserved expression of orthologous genes in specific cell types across the two larvae, such as myocytes, proliferative cells, ciliary band cells and a subset of apical neurons. While this could be seen as an argument for larval homology, similarities in expression of myocytes, proliferative cells and neurons are likely due to cell family homology at the level of metazoa and do not present a strong indication for homology of larvae [35]. On the other hand, ciliary bands cells and apical neurons are likely to be larval specific characters and could indicate a potential homology of these two larvae. Particularly, we detected several TFs as well as spiralian-specific genes co-expressed in the ciliary bands of both larvae. Although exciting, our work also reveals that many shared genes between any given cell types are effector genes (genes coding for structural components, enzymes, etc.) with only a handful of TFs convincingly co-expressed, which leaves open the possibility that similarities recovered are due to convergent evolution and/or that remaining traces of a common origin are scarce. For this reason, generating more high quality scRNA-seq datasets (i.e. with good cell and gene coverage) in spiralian larvae at broader evolutionary distances will be crucial to assess whether these similarities are conserved.

### Single-cell, regeneration and stem cells

One of the few unifying traits of Spiralia is their ability to regenerate (Figure 1); however, regenerative abilities are varied both across and within phyla so it is far from clear whether this represents a real plesiomorphy [36, 37]. For instance, molluscs can only regenerate single organs, whereas most flatworms can regenerate their whole bodies from small fragments and, within annelids, some species can regenerate their body from any segments while others are completely incapable to regenerate [37]. In a way, the distribution and degree of variation of regenerative abilities makes spiralians an interesting group to study the evolution of this character. Still, our understanding of mechanisms and molecular underpinnings of regeneration remains limited for many spiralian clades.

In general, animal regeneration relies either upon a pool of undifferentiated proliferative cells or on the dedifferentiation of pre-existing differentiated cells and both these processes are observed in Spiralia [37, 38]. Populations of proliferating cells deemed responsible for regeneration have been found in both annelids and flatworms and they were historically termed neoblasts. However, within annelids, neoblast *sensu strictu* are only found in clitellates [37]. Interestingly, there has been a focused effort on trying to decipher the molecular signature of neoblast and pluripotent cells in general using single-cell sequencing in several species of both platyhelminthes and annelids (Table 1). In flatworms, pluripotent cells were shown to be a very heterogeneous population containing both undifferentiated cells and progenitors of many differentiated cell types. All pluripotent cells appear to express the canonical marker *piwi*, yet different progenitors are characterised by distinct TFs sets [39]. Similarly, in annelids, authors found an heterogeneous population of cells responsible for posterior growth and agametic reproduction by fission, which express *piwi*, *vasa*, *nanos* and *pumilio* as well as several chromatin regulators together with different cell progenitors, each with their own TF signature [40].

Multipotent cells expressing piwi and vasa, were also recently found in the larva of the polyclad flatworm *Prostheceraeus crozieri* [18]. When the polyclad flatworm atlas was compared to that of planaria (*Schmidtea mediterranea*) these cells aligned well to neoblasts. Moreover, they aligned to proliferative cells of the oyster trochophore larva—located in the differentiating gut. Oyster and flatworm larvae proliferative cells shared the expression of hundreds of genes including *piwi*, *mcm*, *h2a* and *h2b*, *vasa*, *sumo* and the TFs *sox3*, *GTF2B*, *E2F5*, *HES-1* and *ZNF706.*

At first glance, the single-cell datasets currently available reveal a similarity in gene expression between neoblast/proliferative/piwi+ cells across Spiralia; however, an extensive comparison is still lacking. Sampling of more regenerating animals and/or tissues across spiralians could reveal whether similarities are consistent across phyla and allow further comparisons across animals. We note that, the majority of shared genes observed so far across piwi+ cells are part of germ multipotency programs that were likely present in the germline of the last common ancestor of animals and may have been co-opted independently in adults [36].

### The cephalopod brain and visual system

Among spiralians, cephalopods are one of the most fascinating groups as they display a uniquely derived bodyplan underlying extensive organismal novelties. Cephalopods innovations include a crown of prehensile arms and tentacles, a colour shifting skin for camouflage and elaborate nervous system and sensory organs comparable in complexity to that of vertebrates and enabling advanced behaviours [41]. Particularly intriguing is the camera eye of cephalopods, which presents a unique case of convergent evolution with the eye of vertebrates. The two types of eyes markedly differ structurally, for instance in the orientation of the retina towards incoming light [42], but also for the alleged absence of visual information processing neurons in the cephalopod retina (unlike that of vertebrates). Several studies recently used single-cell sequencing to solve that enigma and identify the cell types involved in visual information processing and other neuronal functions in cephalopods [43–46]**.** A key question here is whether neuronal cell types in cephalopods are all newly evolved, and if so, which genes are responsible for their newly acquired identity, or alternatively how many cell types have been inherited from their bilaterian ancestor. Single-cell transcriptomics first showed that few cell types are conserved at long evolutionary distances: photoreceptors show hallmarks of conservation, they share the usage of glutamate as a neurotransmitter with vertebrates, and some evidence suggests the conservation of an ancestral vision-related interneuronal cell type. However, most of the neurons of the optic lobe, hypothesised to have a similar function to the neural retina (by Ramón y Cajal and J.Z. Young), are dopaminergic and seem to constitute novel cell types. At the same time, cephalopod glial cell types show little gene expression conservation with insect or vertebrate glia. Interestingly, cephalopods also possess a richer repertoire of neural genes involved in neurotransmitter synthesis, transport, degradation and reception than both vertebrates and insects, as they often preserved more of the paralogues present in the bilaterian ancestor. These ancient paralogues are readily used to specify their neural cell types, but some lineage-specific gene duplications also enabled the emergence of novel cell types, for example, an opsin duplicate is specifically expressed in an enigmatic photoreceptor cell type [43]. However, gene families that have expanded in cephalopods, such as protocadherins and C2H2 zinc-finger TFs are also expressed in multiple neuronal and non-neural cell types, making it difficult to decipher whether they truly play a key role in establishing cephalopod neuronal diversity [47]. Interestingly, these results also show marked differences between octopus (octopods) and squids (decapods), which have different sets of markers in cholinergic cell types [43, 45]. The case of cephalopods, a group where limited molecular data were available until a few years ago, shows the impact of single-cell sequencing studies to gain molecular insights in new systems and probe organismal novelty. However, characterising vision-related and neural cell types in cephalopod outgroups, such as other molluscs (bivalves, gastropods) will be crucial in understanding how the cephalopod neural cell types evolved.

### Methodological challenges of scRNA-seq in spiralians

In this review, we commented on the most recent scRNA-seq datasets generated in spiralian species, on the impact that this new technology brings to the field of evo-devo, zoology and comparative biology, as well as on possible future directions. However, single-cell information is still lacking for several phyla due to significant methodological challenges that complicate the task of establishing cell type atlases across spiralians (Table 1).

The first challenge in expanding scRNA-seq to more clades is the fact that many spiralian animals are difficult to access and to readily identify taxonomically. Moreover, the vast majority of spiralian clades are marine and raising them in laboratory settings is not always feasible. Some species require travel to remote marine stations which usually lack modern facilities to optimise dissociation protocols and special equipment for scRNA-seq [i.e. fluorescence-activated cell sorting (FACS), 10× chromium controller]. In addition, marine organisms have higher cell osmolarity than classical model organisms for which scRNA-seq protocols have been developed, and this can lead to poor cell viability, low gene count and skewed cell composition (i.e. delicate cell types being depleted from the final dataset). Finally, many spiralian animals are small, which makes gathering enough material in the wild for a single experiment even more challenging. This especially stands for smaller developmental stages such as larvae. For all these reasons, even basic information about mode of development, regenerating abilities and genomic resources are still missing for several clades (Figure 1).

It is hence not surprising that many cell atlases generated so far derive from previously established model organisms, such as the flatworm *Schmidtea mediterranea* and the annelid *Platynereis dumerilii*, as well as of commercially important species such as bivalves [18, 39, 48–50]. Most of these datasets were generated using 10× genomics droplet-based technology whereby cells are isolated into single aqueous droplets inside an oil emulsion using a microfluidic chip (Table 1). Each drop hosts a hydrogel carrying combinatorially barcoded primers which allows the mRNA of different cells to be labelled differently. Once the emulsion is broken the mRNA from different cells can be processed together. Droplet-based methods have the advantage of rapidly capturing a high number of cells, as cells are not sorted into physical wells, which makes them suitable for one-shot experiments for animals that are only available for a short period of time [51]. There are, however, several drawbacks to this method: (i) it requires a large amount of starting material (i.e. cells); (ii) cells cannot be screened nor sorted after capture so it is vital that dissociation is optimised for cell viability; and (iii) 10× controllers needed to load the microfluidic chip are expensive and difficult to transport. A possible solution to these issues is to proceed with fixation of dissociated cells prior to capture so that they can be stored and several batches merged to reach an optimum amount without compromising their viability. A recently developed technique using acetic acid, methanol and glycerol for cell fixation called ACME (a clever re-development of a 19th-century technique called maceration) has been successfully used in several spiralian and marine invertebrates [40, 52, 53]. This technique is usually coupled with a different capture strategy called split-seq which does not rely on any form of cell isolation (microwells, droplets) but rather uses several rounds of combinatorial barcoding; however, FACS sorting is recommended to reduce capture of cellular debris. Split-seq is also cheaper to run than other commercial solutions but setting it up can be costly and time consuming. Another workaround to store and transport animals from remote areas is to perform nuclei extraction on frozen animals which has also been successfully carried out in spiralians and other invertebrates [44, 54].

A further challenge to the expansion of cell type atlases in spiralians is the lack of high-quality genomes or de-novo transcriptomes for several clades (Table 1) that are necessary to map scRNA-seq reads as well as to correctly relate genes across species for comparative purposes. To further complicate things, most single-cell techniques capture the 3′ end of a gene where a three prime untranslated region (3’-UTR) after the translation termination codon is present. These regions are difficult to annotate without extensive transcriptomic resources, and this can result in low proportion of mapped reads. Some workarounds involve adjustment of annotations, particularly as scRNA-seq reads can be used to refine the 3’ UTR annotation in the genome. However, re-annotation is made more challenging by the high level of sequence polymorphism in many marine species [35, 43, 55].

Even when high-quality genomes are present, functional gene annotation in spiralian remains a complex task, because an elevated number of lineage-specific genes (spiralian-specific and/or clade-specific) are uncharacterised due to few model organisms being amenable to genetic studies among spiralians. One notable exception is the model planarian worm *S. mediterranea*, but its fast evolutionary rate complicates homology inference and functional extrapolation to other species. Some of these uncharacterised lineage-specific genes could hold the key to important biological insights. For example, some spiralian specific genes were found in ciliary band of several spiralian marine larvae pointing to the possible homology of ciliary bands and larvae [18, 56].

A final step in many scRNA-seq studies is validation of cell type using *in situ* hybridisation, which makes it possible to visualise the expression of cell type specific markers in the animal of interest. Traditional chromogenic *in situ* hybridisation uses antisense RNA probes labelled with digoxigenin (DIG) that react to alkaline phosphatase (AP) substrates. Several pitfalls of traditional *in situ* hybridisation are, however, to be noted: (i) the process of probe generation requires readily available cDNA; (ii) the protocol is lengthy and can sometimes require a lot of optimisation; and (iii) visualising more than two genes in the same sample is generally not feasible. In recent years, a new technique called hybridisation chain reaction (HCR) was developed to address some of these limitations. HCR allows users to design *in silico* short probes from genes of interest (in the case of scRNA-seq these can be cell type markers) and then carry out hybridisation chain reaction in just 3–5 days [25]. The expression of up to four genes can routinely be imaged using confocal microscopy, with each gene showing fluorescent signals at different wavelengths. The use of spectral imaging and linear unmixing method allows to visualize even more genes in one single experiment [57]. HCR is a valuable tool to validate single-cell atlases: it is quick, does not require RNA extractions to be performed and can be used on multiple genes in combination to accurately characterise cell types [18, 40, 43]. Remaining caveats are that the signal to background ratio of lowly expressed, short genes can be problematic and that probes and reagents are costly, although workaround probe design are available [58].

## DISCUSSION AND OPEN QUESTIONS

We have so far relayed the growing efforts in expanding our knowledge on the least studied bilaterian superclade, Spiralia, and yet so much remains unexplored. Single-cell sequencing provides a powerful tool to vastly enhance our understanding of the biology of many understudied lineages and can hopefully help us solve puzzles that have intrigued evolutionary biology for decades, such as the evolution of larval forms, brains and regeneration.

One of the main challenges when using scRNA-seq to tackle evolutionary questions, and a recurring question throughout our review, is disentangling whether similarity in gene expression is caused by homology or convergent evolution. Some authors have suggested that TFs and gene regulatory networks may be more evolutionary constrained than effector genes and make better candidates when comparing cell type identity across animals. However, we still do not really know to what extent TFs retain a more conserved role than effector genes and recent studies seem to highlight that paralogs are often swapped across lineages—meaning that the expression of paralog genes rather than orthologs is more conserved [34, 35, 59]. In a sense, expanding the availability of scRNA-seq datasets at different evolutionary distances may give us a chance to start testing these ideas and develop new tools to correctly estimate convergence *versus* homology of cell types. Furthermore, another new promising technique is single-cell ATAC-seq, which gives access to the open chromatin regions that include regulatory elements controlling genes at a cellular resolution. By assessing TF binding sites in presumptive regulatory elements, single-cell ATAC-seq enables the reconstruction of the regulatory networks controlling gene expression. Similarity across species of this more elaborate character would represent more compelling evidence for cell type homology [35]. Furthermore, genome alignments could allow detection of conserved non-coding elements informing regulatory foot printing of cell types.

From the seminal studies and examples described in this manuscript, we hope that the application of single-cell transcriptomics will help decipher some long-standing questions in spiralian biology and phylogeny. In fact, several key spiralian characters have not been thoroughly characterised from a molecular perspective, which limits the ability to comment on their homology. The first one is spiral cleavage, a character that, similarly to the trochophore larva, is lost or becomes highly derived in some lineage, for instance cephalopods. Recent studies exploring the molecular mechanism of spiral cleavage and early specification of embryos, showed a conserved role of ERK1/2 [60] but also highlighted the diversity of developmental modes in spiralians. A characterisation of the very determinants of cell specification by directly applying single-cell transcriptomics to the blastomeres would be crucial to further characterise and compare this process across spiralian phyla. Although transcriptomic comparisons suggest that gene expression is most diverse and composed of relatively younger genes at early developmental stages, it is tempting to speculate about the molecular cues of such a conserved developmental process [16].

From a phylogenetic standpoint, several spiralian clades have been hotly debated in molecular analyses but do not present very distinctive characters at the morphological level. For instance, one of the few unifying traits of Gnathifera is the jaw found in rotifers, chaetognaths and gnathostomulids, and one wonders whether conserved cell types are involved in the secretion of these hardened parts [11]. Other proposed spiralian clades would also benefit from the characterisation and comparison of their cell types which could reveal hidden ancestral characters. For instance, the clade Tetraneuralia uniting molluscs and entoprocts has been proposed based on similarities of larval nervous system [61] and subsequently supported by molecular phylogeny [11] (Figure 1). Here, scRNA-seq could help explore the similarities between the nervous system of entoprocts larvae and their putative mollusc counterpart [61, 62]. Moreover, further characterization of cells that make up the lophophore organ of brachiopods, phoronids and ectoprocts could help settle the debate regarding the recently questioned monophyly of lophophorates [10–12]. Finally, a similar reasoning could expand to other clades, such as Vermizoa or Rouphozoa, which remain disputed in phylogenetic studies (Figure 1).

More generally, single-cell transcriptomic comparisons could help shape what the ancestor of Spiralia looked like, what cell types were ancestral to protostomes and what characters instead evolved multiple times. One of the most disputed and yet enthralling hypothesis is the homology of centralised nervous system and other brain structures, and exploring the degree of nervous system centralisation in spiralian ancestors at different nodes as well as their repertory of ancestral cell types would be extremely interesting [63, 64]. Similarly, single-cell transcriptomic could bring new insights into the potential homology or convergent evolution of shells of molluscs and brachiopods [65], as well as other hardened structures (bristles, spicles and chaetae), excretory organs such as protonephridia [66], and blood-like fluid with associated cells in molluscs, annelids or nemerteans.

Overall, the last few years have been an exciting time for spiralian biology as new techniques such as long-read sequencing and single-cell transcriptomics have advanced our understanding of molecular processes beyond traditional models. We hope that single-cell techniques will not only be applied to classical zoological questions such as the evolution of nervous systems, but that this will be an opportunity to appreciate and further study the diversity of organisms and lifestyles in this underappreciated clade.

**Key Points**

- Spiralian clades exhibit a variety of shared traits as well as iconic lineage specific innovations.
- Single-cell sequencing is a powerful tool to rapidly gain cellular and molecular information in lesser studied clades.
- Single-cell sequencing can help unravelling the origin of many spiralian characters, such as larval stages, spiral cleavage, regeneration, shell and brain complexity which remain elusive.

## CREDIT TAXONOMY

Laura Piovani (Conceptualization-Equal, Resources-Equal, Visualization-Equal, Writing—original draft-Equal, Writing—review and editing-Equal), Ferdinand Marlétaz (Conceptualization-Equal, Supervision-Lead, Writing—original draft-Equal, Writing—review and editing-Equal).

## Author Biographies

**Laura Piovani** is a postdoctoral researcher in CLOE at UCL. Her research focuses on applying single-cell sequencing to answer evolutionary questions such as the origin of larval forms.

**Ferdinand Marlétaz** is a senior research fellow in the GEE department at UCL. His focus is the relationship between genome organisations and organismal novelty using functional genomics approaches to uncover regulatory changes.
